# Re-evaluation of the Dutch approach: are recently referred transgender youth different compared to earlier referrals?

**DOI:** 10.1007/s00787-019-01394-6

**Published:** 2019-08-31

**Authors:** Marijn Arnoldussen, Thomas D. Steensma, Arne Popma, Anna I. R. van der Miesen, Jos W. R. Twisk, Annelou L. C. de Vries

**Affiliations:** 1 Department of Child and Adolescent Psychiatry, Center of Expertise on Gender Dysphoria, Amsterdam UMC, Vrije Universiteit Amsterdam, De Boelelaan 1117, Amsterdam, The Netherlands; 2 Department of Epidemiology and Biostatics, Amsterdam UMC, Vrije Universiteit Amsterdam, De Boelelaan 1117, Amsterdam, The Netherlands

**Keywords:** Adolescence, Gender dysphoria, Mental health, Clinical sample, Transgender

## Abstract

The background of this article is to examine whether consecutively transgender clinic-referred adolescents between 2000 and 2016 differ over time in demographic, psychological, diagnostic, and treatment characteristics. The sample under study consisted of 1072 adolescents (404 assigned males, 668 assigned females, mean age 14.6 years, and range 10.1–18.1 years). The data regarding the demographic, diagnostic, and treatment characteristics were collected from the adolescents’ files. Psychological functioning was measured by the Child Behaviour Check List and the Youth Self-Report, intensity of gender dysphoria by the Utrecht Gender Dysphoria Scale. Time trend analyses were performed with 2016 as reference year. Apart from a shift in sex ratio in favour of assigned females, no time trends were observed in demographics and intensity of dysphoria. It was found, however, that the psychological functioning improved somewhat over time (CBCL *β* − 0.396, *p* < 0.001, 95% CI − 0.553 to − 0.240, YSR *β* − 0.278, *p* < 0.001, 95% CI − 0.434 to − 0.122). The percentage of referrals diagnosed with gender dysphoria (mean 84.6%, range 75–97.4%) remained the same. The percentage of diagnosed adolescents that started with affirmative medical treatment (puberty suppression and/or gender-affirming hormones) did not change over time (mean 77.7%; range 53.8–94.9%). These findings suggest that the recently observed exponential increase in referrals might reflect that seeking help for gender dysphoria has become more common rather than that adolescents are referred to gender identity services with lower intensities of gender dysphoria or more psychological difficulties.

## Introduction

Gender dysphoria (GD) is defined as a marked incongruence between a person’s gender identity and the gender assigned at birth accompanied by psychological distress [1]. It is also the term for the diagnostic classification according to the fifth edition of the *Diagnostic and Statistical Manual of Mental Disorders* (DSM-5). Not all people who experience feelings of GD might be diagnosed with DSM-5’s ‘Gender Dysphoria’, because it is likely that they do not all seek assessment and gender affirmative treatment. For this reason and given the lack of systematic epidemiological studies, it is difficult to establish the prevalence of GD. Most former studies on prevalence of GD were based only on the numbers of individuals who are being treated at a transgender clinic. The prevalence in these studies in birth-assigned males ranges from 0.0004 to 0.0352% and in birth-assigned females from 0.0003 to 0.0066% [2]. In recent studies based on self-reported gender identity and GD, higher rates are reported, specifically in adolescents and younger adults [3]. E.g., the Williams institute, a public policy research institute on sexual orientation and gender identity, reports a survey-based prevalence of 0.7% of US adolescents who identify a transgender [4]. Another recent high school sample study that was held amongst 135.760 adolescents under the age of 21 years showed that 0.6% identified as the opposite gender and 3.3% identified as non-binary [5].

While medical gender affirming treatment with hormones and surgeries has been an accepted treatment for adults with GD since the 1970s [6], more reluctance exists concerning medical interventions in adolescents with GD. Some argue that puberty suppression by means of GnRH analogues or affirming hormonal treatment should not be initiated before a person’s physical puberty development is complete, because gender identity still may change during this phase of life, so adolescents should not make decisions regarding this subject [7]. Another point of concern is the lack of data on the long-term physical outcome and the signs from animal models that puberty suppression influences the brain development [8, 9].

Despite these reluctances, the introduction of supposedly fully reversible puberty suppression since the beginning of this century, to provide transgender adolescents who enter puberty with time to explore their gender identity, has rapidly become an accepted and widely prescribed medical intervention for adolescents with GD in Northern America (USA and Canada), and in some countries in Europe and Australia/New-Zealand [10, 11]. Puberty suppression was first introduced as a part of affirming treatment at the Center of Expertise on Gender Dysphoria in Amsterdam in The Netherlands, and therefore, GD treatment that includes puberty suppression is sometimes referred to as the ‘Dutch Model’ [12].

Few evaluative studies of this approach have been performed. Two studies on the first 55 adolescents treated at the Amsterdam clinic showed that behavioural and emotional problems and depressive symptoms decreased, while general functioning improved significantly during puberty suppression [13]. After gender affirming hormonal treatment and surgery, GD was alleviated and psychological functioning further improved [14]. A study on 201 adolescents who attended the transgender clinic in London found that puberty suppression in addition to psychological support was more helpful than psychological support on itself [15].

These results are promising. However, the participants were all adolescents who were referred to transgender clinics before the remarkable recent increase in the number of adolescent referrals [16, 17]. It remains, therefore, unknown whether the positive outcomes of early medical intervention also apply to adolescents who are referred in more recent years. There are several reasons why one could hypothesize that early referrals may differ from more recent registered adolescents. Due to the increased awareness of GD, it could well be that adolescents are referred at a younger age in recent years. In addition, the experimental character of medical treatment may first have attracted the most well-functioning group of adolescents with above average intelligence from stable families with a higher educational background able enough to support their off-spring to ask for a treatment that was still considered controversial in early years. The current flow of referrals might include families that are less well functioning in these aspects. The same may count with regard to co-occurring psychological problems. Finally, present referrals may have less extreme and non-binary forms of GD; a diagnosis of GD could be less likely in present referrals and a smaller percentage would start with medical treatment compared to the early years.

So far, one study has examined whether adolescents (*N* = 203) who were referred to a gender clinic in recent years were different from those in initial reports [18]. In contrast to initial studies, it was found that more birth-assigned females than birth-assigned males applied. Like the earlier studies, high rates of mental health difficulties were reported. Comparisons within the short 2.5 year period (2014–2016) of the first and second half of the adolescents showed that the age at first visit declined somewhat, while the presence of mental health difficulties did not change. Therefore, this study showed some changes over time, but the time span of the study seems too short to actual identify trends. Furthermore, the demographic and the psychological characteristics of the adolescents that were measured were very limited. For example, regarding psychological functioning, they only included the chart-reported mental health history and the Beck Depression Inventory [19].

To gain more insight in the possible change of characteristics of transgender adolescents, we conducted the present study in a large Amsterdam transgender clinic sample (*N* = 1072). The aim of this study is to examine whether there are time trends in demographic, psychological, diagnostic, and treatment characteristics in adolescents referred between 2000 and 2016.

## Methods

### Participants

The 1072 persons included in this study were all consecutively referred adolescents [404 assigned males, 668 assigned females, age 10.14–18.08 years (mean age 14.64, SD 2.19)] who registered at the Center of Expertise on Gender Dysphoria in Amsterdam, The Netherlands, between 2000 and 2016 and could be eligible for medical affirming interventions, including puberty suppression. See Table 1 for further demographic characteristics. Due to missing information, not all the parents’ marital status and parents’ educational level of the adolescents are known.

**Table 1:** Demographic variables

Birth-assigned gender, *N* (%)	
Assigned males at birth	404 (37.7%)
Assigned females at birth	668 (62.3%)
Age at assessment in years, *M *(SD)	14.64 (2.19), range 10.14–18.08
Parents’ marital status, *N *(%)	
Living with both biological parents	579 (54%)
Other	449 (41.9%)
Unknown	44 (4.1%)
Parents’ educational level, *N *(%)	
Vocational educated	581 (54.2%)
Higher vocational and academic educated	322 (30%)
Unknown	169 (15.8%)
Full-scale IQ, *M *(SD)	99.15 (16.08), range 59–145
Diagnosis, *N *(%)	
Not diagnosed with a form of gender dysphoria	57 (5.3%)
Diagnosed with a form of gender dysphoria	908 (84.7%)
Unknown	107 (10%)

*M* mean, *SD* standard deviation

### Procedure

Each adolescent followed the usual assessment, which consisted of several sessions with a psychologist/psychiatrist with the adolescent and the parents together and separate from each other [20]. A psycho-diagnostic assessment was part of the procedure. The recommended treatment plan was provided in a feed-back session.

Some adolescents remained undiagnosed, because the diagnostic process ended prematurely (*N* = 107). In a majority of the cases, the adolescents themselves ended the process due to a discontinued wish for medical treatment. In a minority of the cases, the psychologist decided to prematurely end the diagnostic trajectory due to psychological or social problems that seriously interfered with the diagnostic assessment.

### Demographics

All demographic, psychological, diagnostic, and treatment characteristics were obtained from the adolescents’ files. Four demographic measures were coded: (1) birth-assigned sex of the adolescent, (2) age at assessment, (3) parents’ marital status, and (4) parents’ educational level.

Marital status of the parents was categorized as either “living with both biological parents” or “all other categories” (e.g., single parent*,* divorced, widowed, and adopted). Educational level of the parents was categorized as either “vocational educated” or “higher vocational educated or academic educated”. Traditionally, vocational education is education that focuses on preparing students to work in a trade or a craft, whereas higher vocational education and academic education focuses on higher learning and professional training.

### Measures

#### Intelligence

During the psycho-diagnostic assessment, a Full-Scale IQ was measured by the Dutch version of the Wechsler Intelligence Scale for Children, or the Wechsler Adult Intelligence Scale, depending on the age of the assessed adolescent [21–23].

#### Child Behaviour Checklist and Youth Self-Report

The Child Behaviour Checklist (CBCL) and the Youth Self-Report (YSR) were administered during the diagnostic phase to assess a broad spectrum of behavioural and emotional problems in the adolescents. The CBCL was completed by the respective caregivers and the YSR was completed by the adolescents themselves [24].

Four main outcomes from the CBCL and the YSR were used: (1) the *T*-score for the Total Problem score; (2) the *T*-score for Internalizing problems; (3) the *T*-score for Externalizing problems; (4) the clinical range scores (> 90th percentile) for these three outcomes. The *T*-scores were calculated on the basis of the Dutch norms.

We further calculated the Peer Relation Scale and the Suicidality Scale. As in previous studies, the Peer Relation Scale was created by three items: “Does not get along with other kids” (Item 25), “Gets teased a lot” (Item 38), and “Not liked by other kids” (Item 48) [25]. The Suicidality Scale was based on two items: “Deliberately harms self or attempts suicide" (Item 18) and "Talks about killing self" (Item 91) [26].

#### Utrecht Gender Dysphoria Scale

The Utrecht Gender Dysphoria Scale (UGDS) was administered to measure the intensity of the GD. Answers on the statements can be given on a five-point scale ranging from strongly agree to strongly disagree. There are two different versions for birth-assigned males and females, respectively [27].

### Transgender diagnoses and possible subsequent treatment

For a gender incongruence diagnosis, DSM criteria were followed by the examining psychologist or psychiatrist. Between 2000 and 2014, the DSM-IV-TR was used, and the DSM-5 is used since 2015 [1, 28]. The diagnosis of the adolescent was categorized as either diagnosed with a form of Gender Dysphoria, including Gender Identity Disorder, Gender Identity Disorder Not Otherwise Specified, Gender Dysphoria, Other Specified Gender Dysphoria, and Unspecified Gender Dysphoria or not diagnosed with any form of Gender Dysphoria.

For each participant that was diagnosed with a form of Gender Dysphoria, it was coded whether they started with medical treatment including puberty suppression and/or gender affirming hormones (in older adolescents).

### Statistical analyses

All data analyses were performed using SPSS version 22. A significance level of *p* < 0.05 (two-tailed) was used. Independent *t* tests and Chi-square tests identified if sex assigned at birth was associated with the outcome variables. This variable was included when significant and reported as control variable in the regression analyses.

First, regression analyses with year of assessment as continuous variable were performed and second, if significant, regression analyses with year of assessment as categorical variable represented by dummy variables were carried out. Regarding the regression analyses, for continuous outcomes, linear regression analyses were used, while for the dichotomous outcomes, logistic regression analyses were used.

## Results

### Demographics

**Fig. 1:**
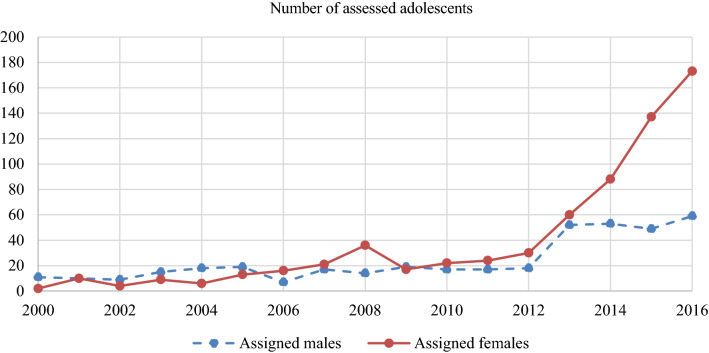
Sex assigned at birth of assessed adolescents

Regression analyses revealed that year of assessment was a significant predictor for sex assigned at birth (OR 1.110, *p* < 0.001, 95% CI 1.078–1.143). Regression analyses with time as a categorical variable also showed this shift in sex ratio, with birth-assigned females being overrepresented in later years compared to earlier years (Fig. 1).

The initial regression analyses regarding age at assessment showed a significant increase over time (*β* 0.061, *p* < 0.001, 95% CI 0.031–0.092), indicating that adolescents who were assessed more recently, were older at the time of intake compared to adolescents who were assessed in previous years. However, the regression analyses with time as a categorical variable showed fluctuations over the years, rather than a trend. Over time, the mean age at intake of the adolescents continued to vary between 14 and 15 years of age. No interaction was found with sex assigned at birth.

Initial analyses showed that the mean Full-Scale IQ of the adolescents became higher over time (*β* 0.366, *p* 0.004, 95% CI 0.116–0.617). Nonetheless, the analyses with time as a categorical variable showed that the mean Full-Scale IQ score of the adolescents remained within the average range each year and that this did not change over time. Our initial regression analyses further showed that most of the adolescents who were assessed at our clinic between 2000 and 2016 lived with both of their biological parents and that this remained the same over the years (OR 0.985, *p* 0.314, 95% CI 0.957–1.014). Furthermore, initial analyses demonstrated that the educational level of the parents of the adolescents increased significantly over time (OR 1.063, *p* < 0.001, 95% CI 1.026–1.100). In the subsequent analyses with time as a categorical variable, however, we found that most of the parents were vocational educated and that this did not change significantly over time. No interaction was found with sex assigned at birth.

### Psychological functioning

**Fig. 2:**
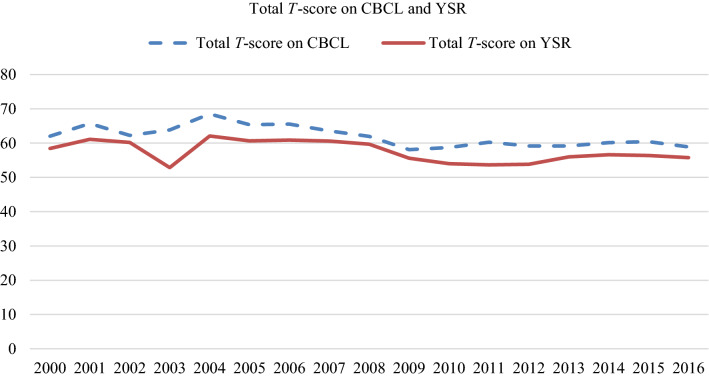
Total *T*-score on CBCL and YSR of assessed adolescents

A decreasing trend was found between 2000 and 2016 in the mean total *T*-score of the assessed adolescents on the CBCL (*β* − 0.396, *p* < 0.001, 95% CI − 0.553 to − 0.240) and the YSR (*β* − 0.278, *p* < 0.001, 95% CI − 0.434 to − 0.122). This decrease in the *T*-score was also found by the analyses with time as a categorical variable for both the CBCL and the YSR. A downward trend is seen until 2008, as shown in Fig. 2. No interactions were found with birth-assigned sex.

Initial analyses showed no trend over time in the mean internalizing total *T*-score on the CBCL (*β* − 0.100, *p* 0.250, 95% CI − 0.272 to 0.071) and the YSR (*β* − 0.011, *p* 0.901, 95% CI − 0.169 to 0.192). No interactions were found with birth-assigned sex.

The mean externalizing total *T*-score on the CBCL and YSR of the adolescents who applied in later years became significantly lower compared to the mean score of the adolescents who applied in early years (CBCL: *β* − 0.408, *p* < 0.001, 95% CI − 0.582 to − 0.234 and YSR: *β* − 0.323, *p* < 0.001, 95% CI − 0.473 to − 0.173). The decreasing trend in the mean externalizing total *T*-score persisted through the analyses with time as a categorical variable. No interactions were found with birth-assigned sex.

Initial analyses showed a significant decrease for the clinical range total *T*-scores on both parent and self-report (CBCL: OR 0.950, *p* 0.002, 95% CI 0.919–0.982 and YSR: OR 0.968, *p* 0.041, 95% CI 0.937–0.999). The more detailed analyses with time as a categorical variable contradicted this trend and showed fluctuation over the years rather than a decrease. No interactions were found with birth-assigned sex.

The clinical range internalizing total *T*-scores of the adolescents on the CBCL and the YSR showed no trend over time (CBCL: OR 0.986, *p* 0.377, 95% CI 0.954–1.018, YSR: OR 1.016, *p* 0.317, 95% CI 0.985–1.049). No interactions were found with birth-assigned sex.

In the clinical range externalizing total *T*-scores, a decreasing trend was found on both parent and self-report (CBCL: OR 0.922, *p* < 0.001, 95% CI 0.892–0.953 and YSR: OR 0.931, *p* < 0.001, 95% CI 0.897–0.966). A similar trend was seen in the subsequent analyses with time as a categorical variable. No interactions were found with birth-assigned sex.

Initial regression analyses showed a decreasing trend in the Peer Relation Scale on the CBCL and the YSR (CBCL: *β* − 0.017, *p* < 0.001, 95% CI − 0.024 to − 0.010 and YSR: *β* − 0.009, *p *0.007, 95% CI − 0.016 to − 0.003), indicating that the quality of the peer relations of adolescents in more recent years was assessed better compared to the quality of the peer relations of adolescents in earlier years. Analyses with time as a categorical variable also showed this trend for the CBCL. On the YSR, however, this decreasing trend was less clear. No interactions were found with sex assigned at birth.

Initial regression analyses showed no trend in time in the score on the Suicidality Scale on the CBCL or the YSR (CBCL: *β* 0.007, *p* 0.273, 95% CI − 0.005 to 0.019 and YSR: *β* 0.006, *p* 0.115, 95% CI − 0.001 to 0.013). Again, no interactions were found with sex assigned at birth.

Linear regression analyses showed that the aforementioned decrease over time of the externalizing problems of the adolescents and the improvement of their peer relationships could explain the decrease in the mean total *T*-score on the CBCL.

### Gender dysphoria intensity

The intensity of the feeling of dysphoria did not change over time for assigned males at birth (*β* 0.055, *p* 0.689, 95% CI − 0.214 to 0.323) and assigned females at birth (*β* − 0.015, *p* 0.835, 95% CI − 0.159 to 0.129).

### Diagnosis and medical treatment

**Fig. 3:**
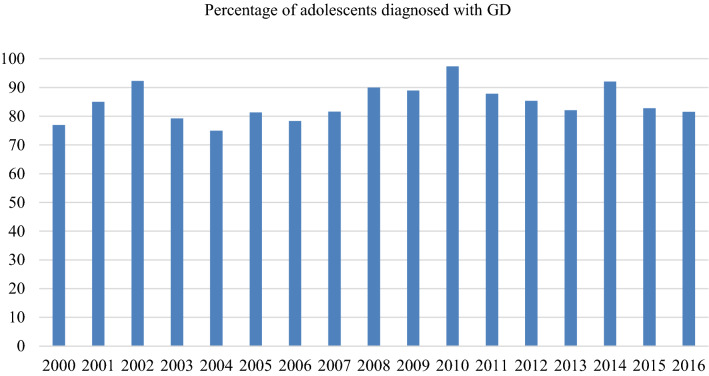
Percentage of assessed adolescents diagnosed with a GD diagnosis

Initial analyses showed a trend over time of an increasing percentage of adolescents who were diagnosed with a form of GD according to the DSM-IV and DSM-5 (OR 1.117, *p* < 0.001, 95% CI 1.060–1.178). In the subsequent analyses, however, we found that most of the adolescents were diagnosed with a form of GD and that this did not change significantly over time (Fig. 3). No interaction was found with sex assigned at birth.

**Fig. 4:**
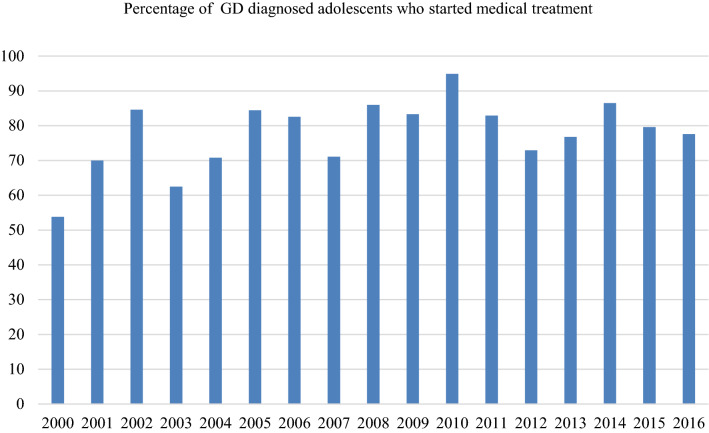
Percentage of adolescents with a GD diagnosis who started with puberty suppression and/or gender affirming hormonal treatment

The initial analyses regarding the actual start of medical treatment after one was diagnosed with GD, showed that over the years, an increasing percentage started with puberty suppression or gender-affirming hormones (OR 1.085, *p* 0.003, 95% CI 1.028–1.146). In the subsequent analyses, instead of a trend, a fluctuation over the years was seen (Fig. 4). No interaction was found with sex assigned at birth.

## Discussion

The present study revealed that despite the sharp increase in the number of adolescent referrals to the Amsterdam transgender clinic in recent years, most demographic and psychological characteristics remained similar over time. Our results also showed that recent referrals were as often diagnosed with GD (mean 84.6%; range 75–97.4%) as early referrals and started just as often with medical treatment including puberty suppression and/or gender affirming hormone treatment (mean 77.7%; range 53.8–94.9%). Despite the fact that GD has become more well known, the mean age at intake of the adolescents did not change over the years. No trends in time were found on the Full-Scale IQ of the adolescents (around 100), parents’ marital status (mostly lived with both biological parents) and parents’ educational level (mostly practically educated). Therefore, contrary to our hypotheses, early referrals seemed just as good as a reflection of the general society as present referrals are.

In addition, although we hypothesized that is was possible that present referrals had more psychological problems, the opposite seems the case. Our analyses showed that psychological functioning of the referred adolescents improved somewhat over time. This could be explained by a subtle improvement in externalizing problems and better peer relations. It might be the case that it has become easier to openly identify as transgender in recent years, so that recent referrals do not have to stand up as fiercely for themselves as earlier referrals. In addition, they seem more accepted by peers.

Regarding the intensity of the experienced GD, our results showed that adolescents who are referred nowadays experience the same high-level of GD as early referrals did. Therefore, contrary again to what was expected, the percentage of adolescents who were diagnosed with a GD diagnosis did not change over time nor did the percentage of GD diagnosed adolescents who started medical treatment. From this, one might conclude that adolescents who are being referred nowadays are still looking for medical treatment, rather than support and counselling only.

The described findings have clinical implications for providing early medical interventions. Since the assessed adolescents are so similar on most relevant characteristics over the years, this provides confidence that early medical treatment may also be helpful for recent referrals. It is likely that previously found results regarding the effectiveness of the Dutch protocol that includes puberty suppression as part of a multidisciplinary approach [13, 14], can be generalized to the transgender adolescents who currently apply.

Next to our results regarding our hypotheses, in our demographic analyses, there was one changing trend. Although in early years, more assigned boys were referred to gender identity services [29], referred adolescents favour birth-assigned females since the mid-2000s [16, 30]. Our study showed that this shift in sex ratio is further skewed. In 2016, the ratio between birth-assigned males and birth-assigned females was 1:2.93, while in 2015 and 2014, it was 1:2.80 and 1:1.66, respectively. One suggested theory to explain this shift is that it is easier for birth-assigned females to be open about their transgender feelings, since they experience less stigma when they behave masculine than birth-assigned males who behave in a more feminine manner [16, 31–33]. Another possible factor that could play a role in this shift is the difference in pubertal onset between birth-assigned females and birth-assigned males. On average, puberty starts earlier in birth-assigned females than it does in birth-assigned males, which could mean that the feelings of dysphoria with one’s body and physical characteristics intensify earlier in birth-assigned females [16]. However, if this hypothesis was correct, one would expect that birth-assigned females would be referred at a younger age than birth-assigned males, and from our results, it appears that the mean age at intake did not differ between these two sexes.

Our study also provides a new insight into factors that have possibly contributed to the recent increase in the number of adolescent referrals in gender identity services. Since most characteristics remained similar, we suggest that GD might be more common than previously thought and the exponential increase in referrals is just a reflection thereof. The increased publicity and visibility may have helped more young people and their parents to recognize and come out for their transgender feelings, and they seem more likely to dare to seek assessment and treatment.

Our results should be seen in the light of some limitations. First of all, the adolescents in this study are part of a clinic-referred sample. Therefore, the drawn conclusions cannot be generalized to transgender identifying youth from the general population. It could be that outside of our gender identity clinic, changes over time have taken place that we did not observe. Second, some relevant characteristics could not be included in this study since data collection started many years ago when awareness lacked on these. For example, if the identity of the adolescents is exclusively masculine or feminine or that, e.g., non-binary, agender, or gender fluid, would better define their identity. The UGDS is not an ideal instrument to detect these gender identities. In the third place, there may be trends in recent years that we could not yet demonstrate with the data we have. Because the increase in referrals may reflect a catch-up of adolescents experiencing GD, it is likely that this increase will come to a plateau when the catch-up is complete. We would have to repeat the analyses at that point in time to identify any other trends. Finally, this study is a historical cohort study. Thus, we can describe the longitudinal trends we see, but we can only speculate about the causes for these trends.

## Conclusion

Our study assured that adolescents referred to a long existing specialized transgender service did not show critical changes in key demographic, psychological, diagnostic, and treatment characteristics over 16 years with the exception of a shift in sex ratio. This may suggest that in the early years, only the tip of the iceberg of the actual number of transgender youth was presented to a transgender clinic and this iceberg has come to surface in recent years. In other words, it seems to be that the increase in the number of referrals is probably due to the fact that feelings of GD are more common than originally expected, rather than that the threshold to register at a transgender clinic has decreased in such extent, that a group of other adolescents is seen nowadays. This finding suggests that a larger group of adolescents who experience GD is able to profit from gender affirming treatment, including puberty suppression.
